# Delayed diagnosis and genetic testing in spinal muscular atrophy: a case series

**DOI:** 10.1080/07853890.2026.2728198

**Published:** 2026-09-06

**Authors:** Xihua Guo, Yuqi Luo, Mingchun Li, Chunguang Li, Zihan Chang, Shuzhen Zhu, Qing Wang, Yanjun Huang

**Affiliations:** Department of Neurology, Zhujiang Hospital of Southern Medical University, Guangzhou, P.R. China

**Keywords:** Spinal muscular atrophy, clinical characterization, delayed diagnosis, disease modifying treatment

## Abstract

**Background:**

Spinal muscular atrophy (SMA) is a recessively inherited autosomal neuromuscular disorder that is associated with deletions or disease-causing variants in the survival motor neuron 1 (*SMN1*) gene. Delayed diagnosis of SMA remains a common issue worldwide, particularly in regions with unequal medical resources.

**Case presentation:**

Patient 1 was a 21-year-old man who had experienced limb weakness and muscle atrophy for 20 years. At the age of 6, he underwent genetic testing and was diagnosed with SMA type II. Patient 2 was a 26-year-old man who had been complaining of progressive limb weakness for 11 years and muscle atrophy for 5 years. When he was 20 years old, he experienced muscle atrophy of both legs and was diagnosed with SMA type III after genetic testing. Patient 3 was a 40-year-old man who presented with slowly progressive lower limb weakness since the age of 15. He was misdiagnosed with Duchenne muscular dystrophy in age 20. He was referred to our clinic at the age 40 and was ultimately confirmed to have SMA after genetic testing. Patients 4 and 5 were sisters, who complained of lower limb weakness and were recently diagnosed with SMA.

**Conclusions:**

This case series highlights the current status and possible reasons for delayed diagnosis and delayed initiation of treatment for SMA, including limited awareness of SMA, low accessibility of genetic testing, and uneven distribution of medical resources.

## Introduction

Spinal muscular atrophy (SMA) is a neuromuscular disorder characterized by the degeneration of anterior horn cells in the human spinal cord and subsequent loss of function in motor neurons, thus leading to progressive muscular atrophy, muscle weakness, and muscle paralysis. According to the age of onset and maximum achieved motor function, SMA is classified into the following four clinical types: type I (weak infants unable to sit unsupported); type II (patients able to sit independently but unable to stand or walk); type III (patients who are ambulant from childhood); and type IV (adult onset) [1,2]. The gold standard for the diagnosis of spinal muscular atrophy is genetic testing, with additional clinical classification, electrophysiological findings, and imaging evidence serving as supportive examinations. Since 2016, three therapies have been available for the treatment of SMA: the antisense oligonucleotide known as nusinersen, the gene replacement therapy known as onasemnogene abeparvovec, and the small molecule splicing modifier known as risdiplam. These agents have been shown to improve both survival and quality of life in patients with SMA. Nusinersen was launched in China in 2019 and risdiplam was launched in China in 2021. However, early diagnosis and treatment of SMA in China remain challenging, and are constrained by low public disease awareness, regional disparities in healthcare access, and the absence of standardized genetic testing methods. Here, we reported five cases of SMA patients who experienced delayed diagnosis or delayed treatment due to various factors, highlighting the importance of genetic diagnosis.

## Case presentation

### Ethical approval statement

This study was approved by the Ethics Committee of Zhujiang Hospital, Southern Medical University, Guangzhou, China (2026-KY-061-01) and was conducted in accordance with the Declaration of Helsinki. Written informed consents for publication of the clinical data were obtained from all individuals.

### Case 1

The first case involved a 21-year-old man. When the patient was approximately one year old, his family found that his motor development was delayed compared with that of his peers. He could not walk and had difficulty completing movements, such as lifting, stretching and holding of both upper limbs. He was able to sit independently. When he was 6 years old, he first visited the hospital. A genetic test (PCR-melting curve analysis) was conducted in 2009, and the results revealed a homozygous deletion of exons 7 and 8 of the *SMN1* gene. He was subsequently diagnosed with SMA type II and was given symptomatic treatment. As the disease progressed, limb weakness gradually worsened. At the age of 20, he experienced difficulty chewing, occasional choking, dysphagia, and weight loss in 2023. Therefore, he visited the hospital for further treatment and underwent lumbar punctures and was intrathecally administered 12 mg of nusinersen sodium injection for four doses. The patient’s cousin experienced a similar disease.

At the age of 21, he was admitted to our hospital in 2024. Our physical examinations revealed that the patient was unable to stand independently and was sitting in a chair; his upper arms could not be raised upward, and his lower limbs could not move independently. In addition, his tongue muscles, bilateral trapezius muscles, masseter muscles, upper limb muscles, and lower limb muscles were atrophied. Given the convenience and compliance of treatment the patient received risdiplam, which was approved to treat SMA in China in 2021.

### Case 2

Case 2 involved a 26-year-old man. When the patient was 15 years old, he found for the first time that his leg motor function was worse than that of his peers. At that time, he had difficulty squatting, and his upper limbs exhibited postural and behavioral tremors. At the age of 20, he experienced muscle atrophy primarily affected the bilateral thigh muscles. So he visited the hospital and was diagnosed with type 3 spinal muscular atrophy after genetic testing (PCR-melting curve analysis) in 2019, which revealed deletions of exons 7 and 8 of the *SMN1* gene. He denied any family history of hereditary muscular disorders. The patient previously had a pituitary tumor. By 2024, the patient had undergone lumbar punctures and was intrathecally administered 12 mg of nusinersen sodium for a total of eight doses.

To continue treatment, the patient was admitted to our hospital in 2024. On physical examination, he had limb weakness and could not stand up independently after squatting. In addition, his bilateral trapezius muscles, sternocleidomastoid muscles, upper limb muscles and lower limb muscles all exhibited muscular atrophy. During this hospitalization in 2024, considering the convenience and compliance of treatment, the patient was treated with risdiplam, a specific anti-SMA drug.

### Case 3

Case 3 involved a 40-year-old man. At the age of 15, the patient complained of lower limb weakness, and he was prone to falling and experienced fatigue in his legs after exercising. He had no family history of hereditary muscle weakness and denied any other past medical history. During the disease, the patient experienced muscle atrophy in the proximal extremities that progressively worsened and pseudohypertrophy in the gastrocnemius muscle. At age 20, he sought treatment at a hospital in 2004. A muscle biopsy revealed muscle fiber hypertrophy, resulting in a diagnosis of Duchenne muscular dystrophy. There was no effective treatment at that time, so the patient did not receive any treatment.

**Figure 1.**
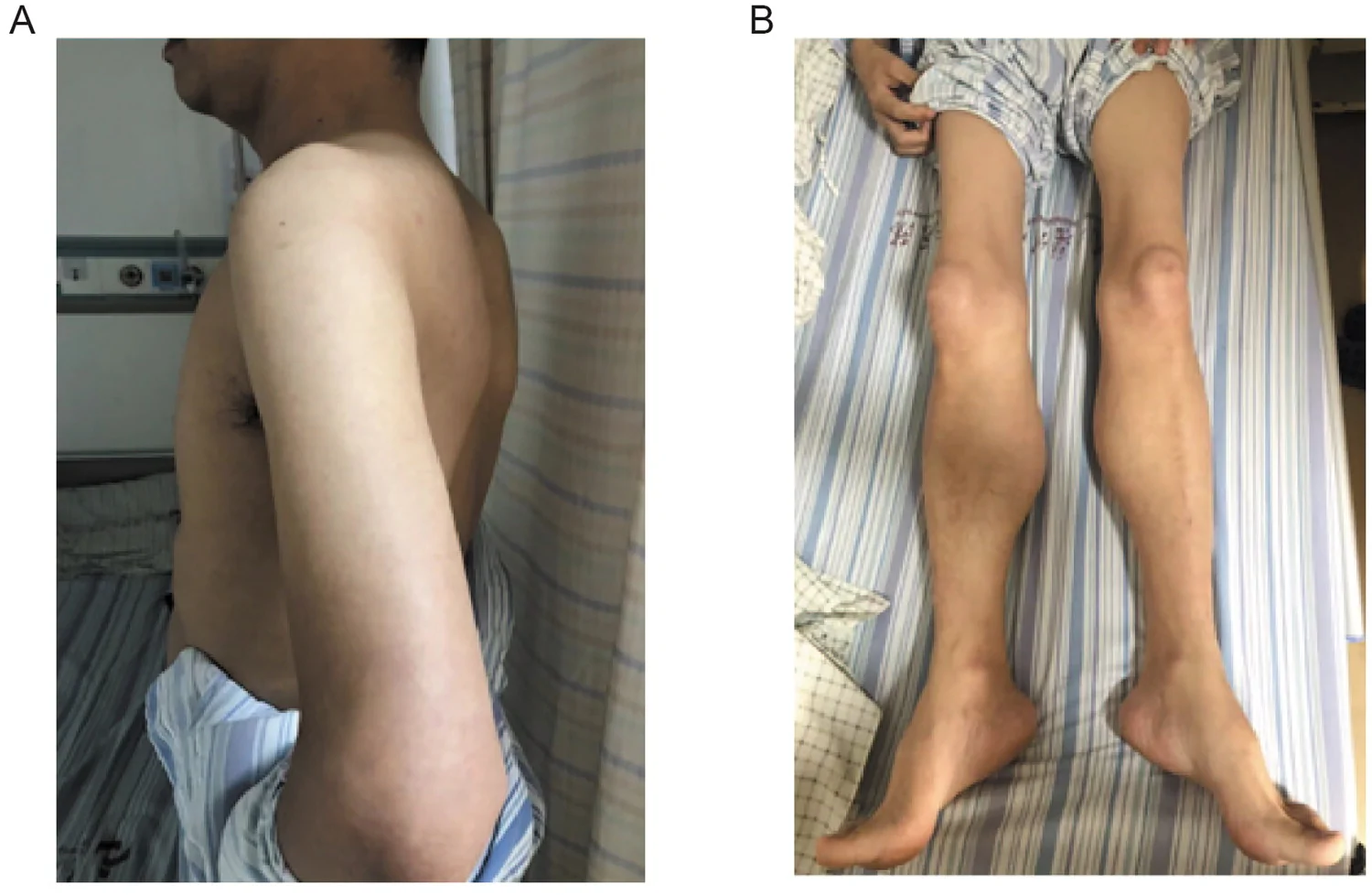
Current clinical condition of case 3. (A) The patient’s proximal muscles in the upper and lower limbs were significantly atrophied and weakened; (B) Pseudohypertrophy of the gastrocnemius muscles in the lower extremities. Permission was obtained from the patient to be published for these photos for research purposes.

**Figure 2.**
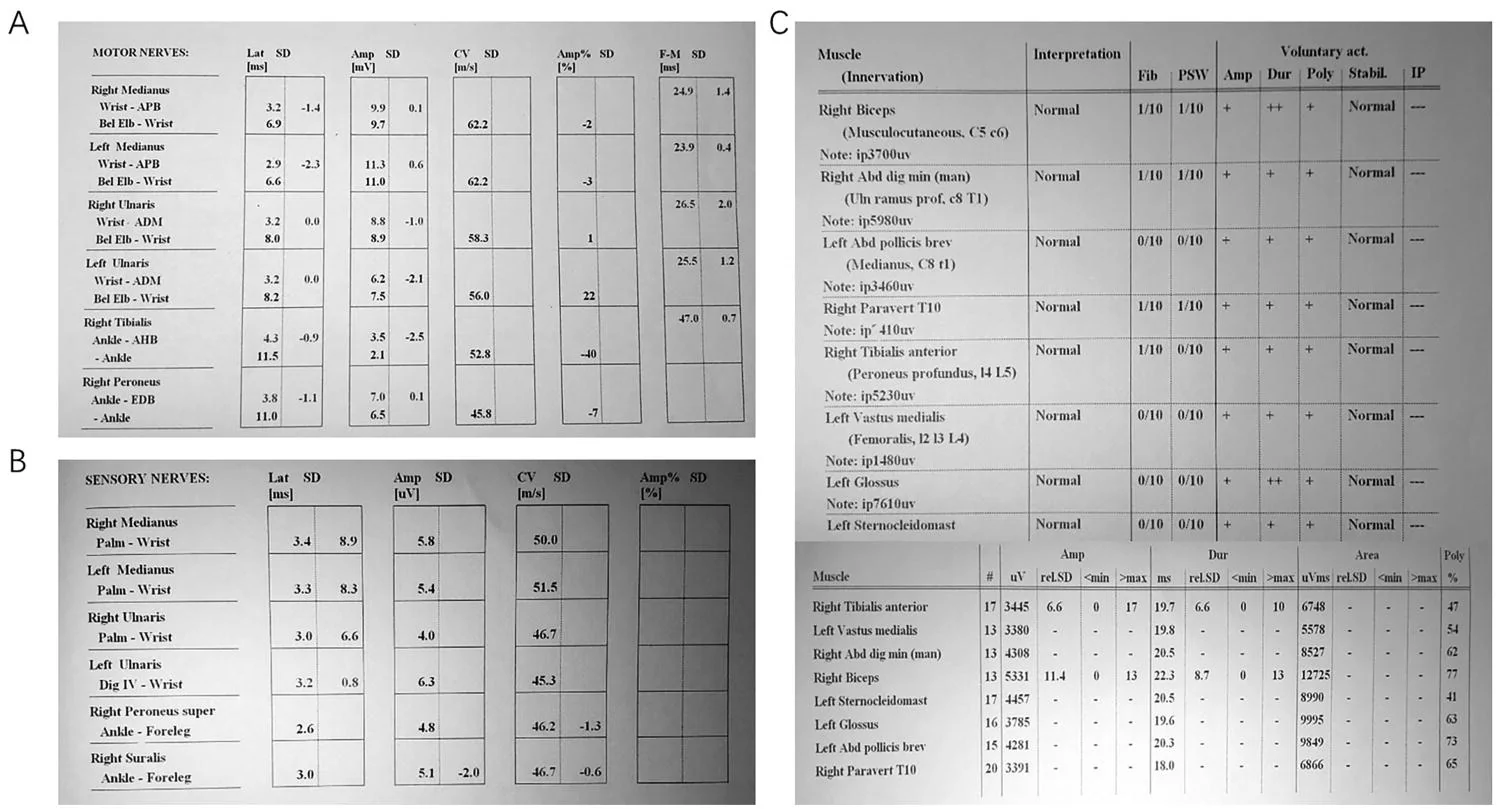
Electromyography results of case 3. (A) Motor nerves conduction velocity: the CMAP amplitude in the tibial nerve was decreased; (B) Sensory nerves conduction velocity: normal. (C) MUAP (motor unit action potential) Spontaneous activity: abnormal muscle fiber potentials seen as fibrillatory potentials and positive sharp waves; motor unit potentials had prolonged and increased wave amplitudes; polyphase wave: increased, issued at a simple phase frequency.

**Figure 3.**
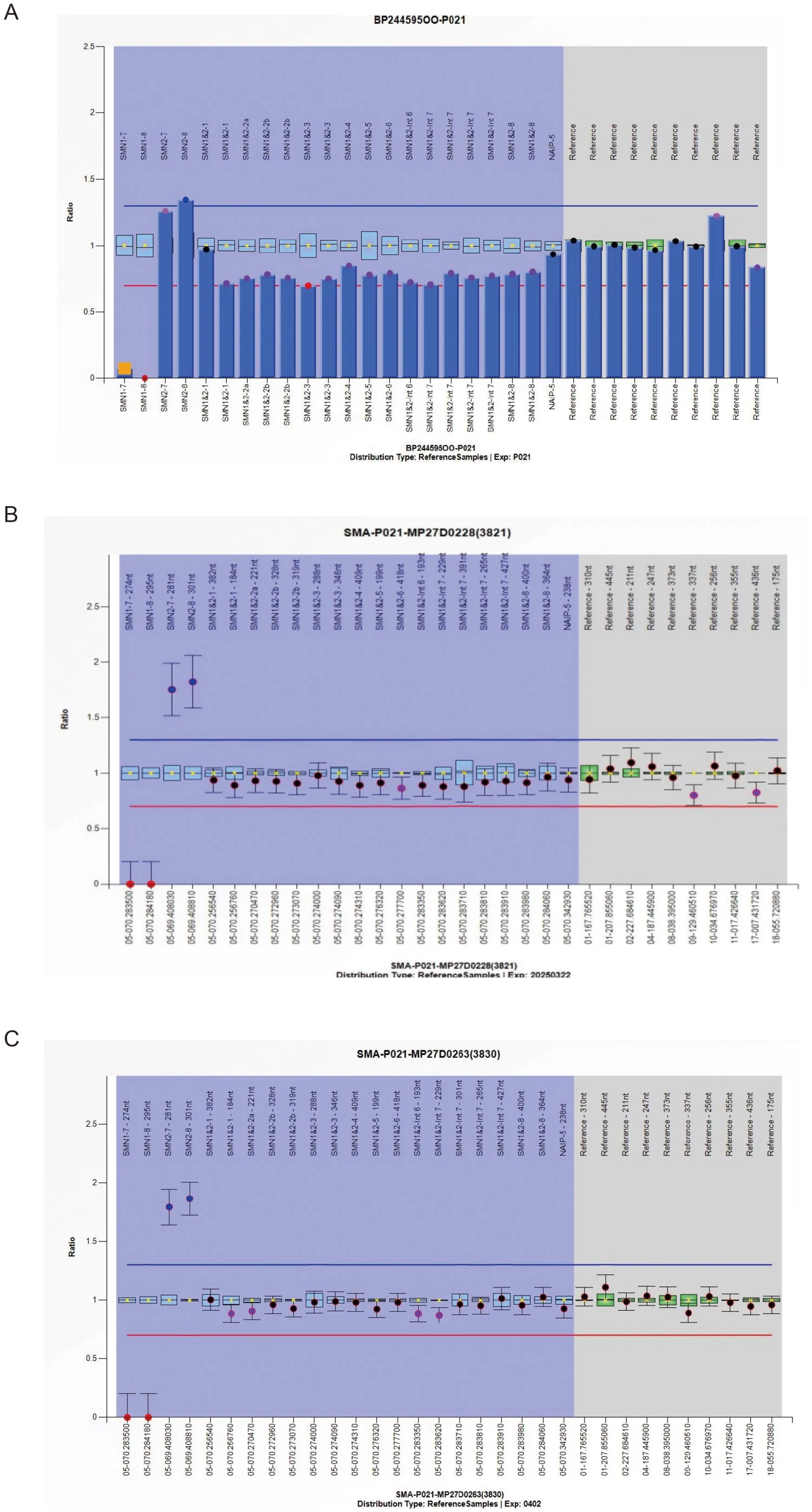
Genetic testing of *SMN1* gene and *SMN2* gene of case 3, 4, and 5. (A) The results showed a deletion of exon 7 in the *SMN1* gene and three copies of the *SMN2* gene. (B) The results showed deletion of exon 7 and 8 in the SMN1 gene and four copies of exon 7/8 in the *SMN2* gene. (C) The results showed deletion of exon 7 and 8 in the SMN1 gene and four copies of exon 7/8 in the *SMN2* gene.

At age 40, the patient was admitted to our hospital for further evaluation in 2024. He had limb weakness, with slightly decreased strength in the upper limbs and markedly decreased strength in the lower limbs (Figure 1). The patient remains capable of slow walking and can perform some activities of daily living. On physical examination, cranial nerve function and sensory functions were intact and cerebellar signs and Gower’s signs were absent. Laboratory tests revealed mildly elevated creatine kinase levels. A nerve conduction study (NCS) revealed a reduced compound muscle action potential (CMAP) in the right tibial nerve, and needle electromyography demonstrated neurogenic damage (Figure 2). A muscle biopsy of the gastrocnemius muscle was performed and revealed neurogenic skeletal muscle damage. In addition, genetic testing (PCR-melting curve analysis) revealed a deletion of exon 7 in the *SMN1* gene and three copies of the *SMN2* gene (Figure 3(A)), confirming the diagnosis of SMA. With the patient’s consent and considering the convenience and compliance of treatment, he was treated with risdiplam.

### Case 4

**Figure 4.**
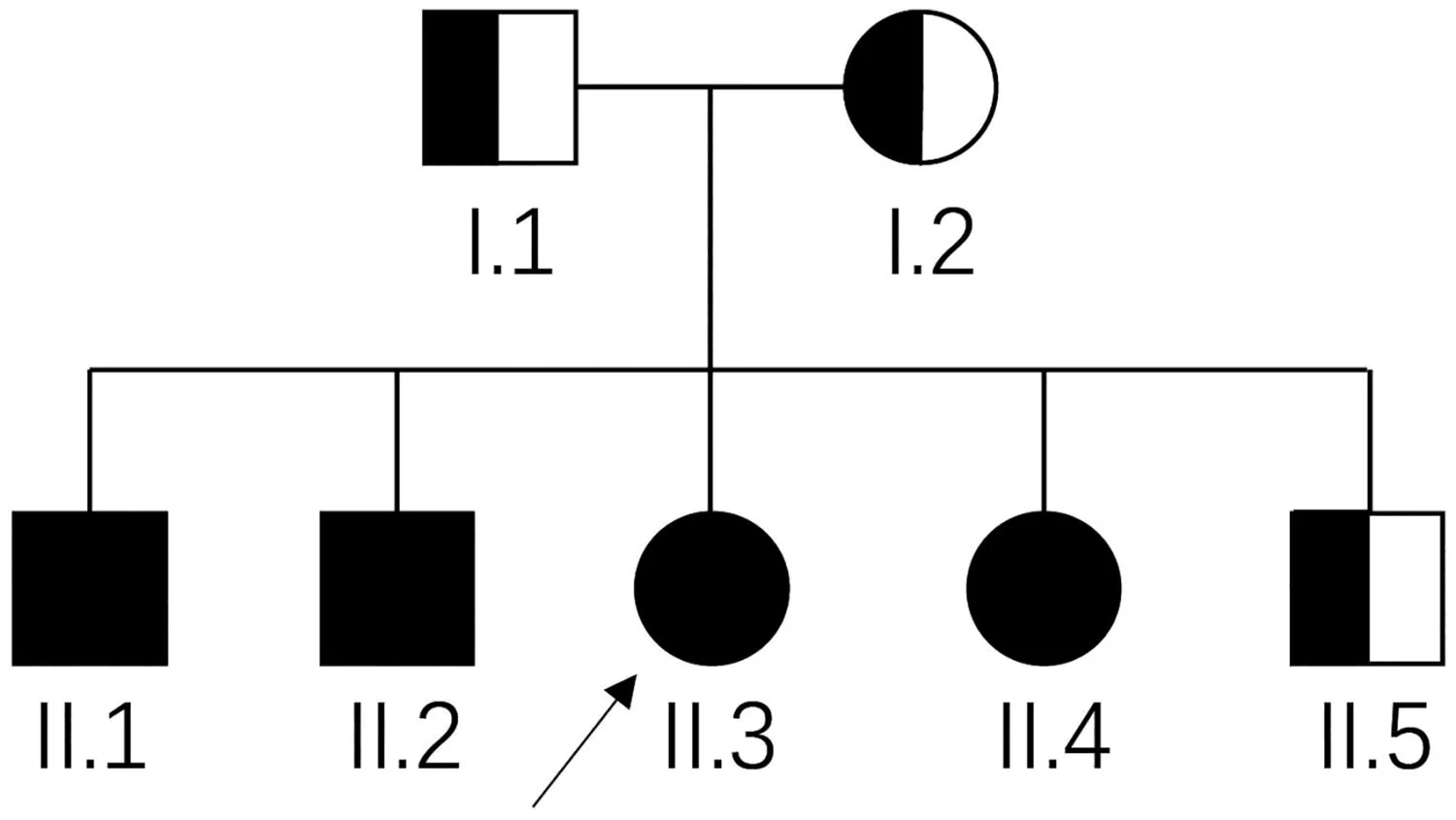
Families’ pedigree of case 4 and 5. Roman numerals represent the generations. Arabic numerals identify individuals. Arrows indicate the probands. II.3 represents case 4, and II.4 represents case 5.

Case 4 involed a 26-year-old woman. At the age of 6, her lower limb weakness started, and the motor function of both legs gradually decreased. She was unable to participate in sports. Neither family members nor patients take this symptom seriously. During the disease process, the patient had not received any treatment, but she was still able to walk slowly and perform some daily activities. In her family, her two brothers and one sister (referred to as case 5) all exhibited similar symptoms (Figure 4). At age 26, she underwent a genetic test (PCR-melting curve analysis), which revealed a deletion of exon 7 in the *SMN1* gene and four copies of exons 7 and 8 in the *SMN2* gene (Figure 3(B)), and was diagnosed with SMA type III.

For further treatment, both the patient and her sister (case 5) were admitted to our hospital in 2025. Physical examination revealed that patient’s bilateral lower limb muscles exhibited muscular atrophy. However, she was able to walk slowly. Following the patient’s consent and considering treatment convenience and adherence, risdiplam was administered. Additionally, the two brothers declined participation, citing the prohibitive distance from the research center and financial constraints as the main reasons.

### Case 5

Case 5 involved a 23-year-old woman. When she was 3 years old, her lower limb weakness started, the motor function of both legs gradually decreased. Furthermore, the weakness of her lower limbs gradually worsened. Neither family members nor patients take this symptom seriously. At the age of 5, she could not stand or walk independently. Two of her brothers and one sister (case 4) exhibited similar symptoms. The patient had not received any treatment. After the initial evaluation, the patient was admitted to our hospital for additional treatment in 2025. A genetic test (PCR-melting curve analysis) revealed a deletion of exon 7 in the *SMN1* gene and four copies of exons 7 and 8 in the *SMN2* gene (Figure 3(C)). Thus, she was diagnosed with type III SMA at age 23. Physical examination revealed that patient’s bilateral lower limb muscles exhibited muscular atrophy. The patients could only sit independently and could not stand alone. The results of needle electromyography suggested the presence of neurogenic damage. With the patient’s consent and considering the convenience and compliance of treatment she was treated with risdiplam.

## Discussion

The worldwide incidence of SMA is approximately 1 in 10,000 live births. The sex ratio of patients is approximately equal between males and females [3]. Among our patients, three were males. The initial symptom observed in all three patients was limb weakness. All of the SMA patients in our case series experienced delayed diagnosis or delayed disease-modifying treatment (DMT) initiation. In some cases, misdiagnosis persisted for more than ten years (Table 1). The underlying reasons possibly include the easily confusable clinical manifestations, overlapping symptoms, limited accessibility of genetic diagnostic technology, and uneven distribution of healthcare resources.

**Table 1.** Summary of main clinical findings and biochemical examination of the five reported cases.

	Case 1	Case 2	Case 3	Case 4	Case 5
Clinical diagnosis	SMA type II	SMA type III	SMA type III	SMA type III	SMA type III
Sex	male	male	male	female	Female
Age at symptom onset, years	1	15	15	6	3
Age at genetic diagnosis, years	6(2009)	20(2019)	40(2024)	26(2025)	23(2025)
Age at DMT initiation, year	20(2023)	24(2023)	40(2024)	26(2025)	23(2025)
Family history	Patient’s cousin	**-**	**-**	Two brothers and one sister	Two brothers and one sister
Initial symptoms	Limb weakness and muscle atrophy	Lower limbs weakness	Lower limbs weakness	Lower limbs weakness	Lower limbs weakness
Pseudomuscular hypertrophy	−	−	+	−	−
Postural and behavioral tremor	−	+	−	−	−
Walk independently	−	+	+	+	−
Chewing problem	+	−	−	−	−
Choking	+	−	−	−	−
Dysphagia	+	−	−	−	−
CK plasma levels (IU/L)	−	1418.4	734.1	177	235.3
Gene mutation site	Deletion of exons 7 and 8 of *SMN1* gene	Deletion of exon 7 and 8 of *SMN1* gene and 4 copies of exon 7, 3 copies of exon 8 in the *SMN2* gene	Deletion of exon 7 and 8 in the *SMN1* gene and 3 copies of exon 7/8 in the *SMN2* gene	Deletion of exon 7 and 8 in the *SMN1* gene and 4 copies of exon 7/8 in the *SMN2* gene	Deletion of exon 7 and 8 in the *SMN1* gene and 4 copies of exon 7/8 in the *SMN2* gene

*Note*. DMT: disease modifying treatment. *SMN2* copy number in case 1 was not measured.

As illustrated in Cases 3–5, the delay in diagnosing SMA was primarily attributable to inadequate public awareness of the disease and the limited accessibility of genetic testing in China. In addition, when the patient in case 1 was 20 years old, he complained about dysphagia and choking, which eventually led to weight loss and malnutrition. Recently, approximately 60% of 146 children and adolescents with SMA II were reported to have feeding difficulties, including eating problems, choking, chewing problems and/or swallowing issues (including aspiration), prolonged meal times, or weight loss [4,5]. The main complications of feeding difficulties are aspiration pneumonia and malnutrition. Initial signs in children with SMA II appear before 18 months of age and manifest as a lag or delay in motor and postural skills, which is associated with axial and segmental hypotonia [5,6].

In Case 3, the patient’s early-adult onset was initially misdiagnosed as Duchenne muscular dystrophy, which was mainly due to the mild clinical symptoms, limited healthcare resources, a lack of standardized rare disease protocols, and the unavailability of *SMN1* gene testing in China at the time of onset. The age of onset of SMA III is approximately 18 months or older, with a mean age of onset of approximately 39 months [7]. The initial symptoms include muscle weakness and atrophy that begin in the proximal muscles of the lower limbs and later ascend to the upper limbs and trunk. As the disease progresses, pelvic muscle involvement leads to abnormal gait, but patients with SMA III usually have few symptoms of respiratory muscle weakness. The clinical manifestations of children with SMA III are similar to those of children with muscular dystrophy; thus, misdiagnosis is a common event. A prospective study [8] explored the natural history of SMA type II and SMA type III, categorizing patients into four groups based on the maximum achieved function: walking independently (SMA IIIb), walking with assistance (SMA IIIa), sitting independently (SMA II), and sitting with support (SMA I). In patients with SMA IIIb and more than 2 years age at onset, walking was maintained until a median age of 44 years.

Previously, electromyography (EMG) and muscle biopsies were instrumental in determining the diagnosis of SMA [3]. Currently, genetic testing is the gold standard for SMA diagnosis, due to the fact that approximately 95% of SMA patients exhibit homozygous deletions of exons 7 and 8, or deletions of exon 7 alone in the *SMN1* gene. In patient 3, due to the slow progression of SMA type III, some typical clinical manifestations were not detected early, leading to a delayed diagnosis or even misdiagnosis [9]. Cases 4 and 5 are sisters, and there are brothers in the same family with similar symptoms. Their symptoms appeared at an early age, and the delayed diagnosis was largely attributed to an uneven distribution of medical resources, an inadequate tiered diagnosis and treatment system, and significant financial burdens. Additionally, the consecutive occurrence of SMA in multiple children in this family notably reflects both the public’s limited awareness of genetic disorders and the lack of universal coverage of SMA in newborn screening programs. To date, many genetic diseases in developing countries and regions are often overlooked because of the challenges associated with diagnosis and limited treatment availability. In four of the cases presented here, genetic testing was performed after 2019 (Table 1), which reflects the delayed availability and limited accessibility of SMA genetic testing in China. For the diagnosis of rare hereditary neuromuscular disorders, clinicians should pay more attention to the clinical details of the patient’s history and physical examinations to refine the differential diagnosis.

Currently, the only approved DMT for SMA includes nusinersen, zolgensma, and risdiplam, which became clinically available around approximately 2016–2017. As an antisense oligonucleotide that modulates SMN2 splicing, nusinersen was the first SMN replacement therapy to achieve regulatory approval. Clinical trials of nusinersen have demonstrated significant improvements in long-term safety and efficacy in patients with SMA [10]. A previous study of adult patients treated with nusinersen showed that half of the patients reported subjective functional improvement, but also identified potential adverse effects, including post lumbar puncture headache and urinary incontinence following laminectomy [11]. Additionally, risdiplam, which is the first orally administered drug for SMA, provides a convenient and less painful treatment option. As an *SMN2* mRNA splicing modifier, risdiplam regulates the splicing of the *SMN2* gene (homologous gene of *SMN1*) through double-point specificity while promoting the inclusion of exon 7 during *SMN2* mRNA transcription, thereby increasing the expression of the SMN protein [12]. Both domestic and international clinical treatment guidelines, as well as expert consensus, indicate that initiating treatment at an earlier stage yields greater benefits [13,14]. In China, nusinersen was approved for clinical use in 2019; however, its initial price was prohibitively high, placing it out of reach for most families. This situation was substantially improved ed in 2021, when the drug was included in the National Reimbursement Drug List (NRDL), resulting in a marked reduction in patient out-of-pocket expenses. Meanwhile, risdiplam was launched in China in 2021 and was included in the NRDL the same year. Despite these therapeutic advances, the patients in Cases 1 and 2 remained untreated following their SMA diagnosis, as DMT had not yet been made available or reimbursed in China at the time of their diagnosis. Therefore, the delayed availability and high cost of SMA therapies have posed substantial challenges for disease management in China.

However, several limitations of this study should be acknowledged. First, the sample size was insufficient to adequately characterize rare clinical features of the disease. Second, the follow-up duration was insufficient to evaluate long-term efficacy.

## Conclusion

Delayed diagnosis of SMA and delayed initiation of DMT remain common events in China, which are possibly driven by low public awareness of genetic disorders, inadequate clinical standards for genetic diseases, and poor access to genetic testing.

## Supplementary Material

CARE checklist English.pdf

## Data Availability

The data that support the findings of this study are available from the corresponding author, Qing Wang, upon reasonable request.
