# Supplementary material for: Delayed diagnosis and genetic testing in spinal muscular atrophy: a case series
Source: Ann Med. 2026 Sep 6;58(1):2728198. doi: 10.1080/07853890.2026.2728198 (PMC13552307; doi:10.1080/07853890.2026.2728198)
Supplement: Supplementary file 1 [file IANN_A_2728198_SM4394.pdf]

CARE case report guidelines

CARE Checklist of information to include when writing a case report

(cc) BY-NC-ND

Topic	Item	Checklist item description	Reported on Line
Title	1	The diagnosis or intervention of primary focus followed by the words "case report"	Page 1
Key Words	2	2 to 5 key words that identify diagnoses or interventions in this case report, including "case report"	Page 3
Abstract (no references)	3a	Introduction: What is unique about this case and what does it add to the scientific literature?	Page 3
	3b	Main symptoms and/or important clinical findings	Page 3
	3c	The main diagnoses, therapeutic interventions, and outcomes	Page 3
	3d	Conclusion—What is the main "take-away" lesson(s) from this case?	Page 3
Introduction	4	One or two paragraphs summarizing why this case is unique (**may include references**)	Page 4
Patient Information	5a	De-identified patient specific information.	Page 4-7
	5b	Primary concerns and symptoms of the patient.	Page 4-7
	5c	Medical, family, and psycho-social history including relevant genetic information	Page 4-7
	5d	Relevant past interventions with outcomes	Page 4-7
Clinical Findings	6	Describe significant physical examination (PE) and important clinical findings.	Page 4-7
Timeline	7	Historical and current information from this episode of care organized as a timeline	Page 4-7
Diagnostic Assessment	8a	Diagnostic testing (such as PE, laboratory testing, imaging, surveys).	Page 4-7
	8b	Diagnostic challenges (such as access to testing, financial, or cultural)	Page 4-7
	8c	Diagnosis (including other diagnoses considered)	Page 4-7
	8d	Prognosis (such as staging in oncology) where applicable	Page 4-7
Therapeutic Intervention	9a	Types of therapeutic intervention (such as pharmacologic, surgical, preventive, self-care)	Page 4-7
	9b	Administration of therapeutic intervention (such as dosage, strength, duration)	Page 4-7
	9c	Changes in therapeutic intervention (with rationale)	Page 4-7
Follow-up and Outcomes	10a	Clinician and patient-assessed outcomes (if available)	N/A
	10b	Important follow-up diagnostic and other test results	Page 6-7
	10c	Intervention adherence and tolerability (How was this assessed?)	N/A
	10d	Adverse and unanticipated events	N/A
Discussion	11a	A scientific discussion of the strengths AND limitations associated with this case report	Page 8-10
	11b	Discussion of the relevant medical literature **with references**.	Page 8-11
	11c	The scientific rationale for any conclusions (including assessment of possible causes)	Page 9-11
	11d	The primary "take-away" lessons of this case report (without references) in a one paragraph conclusion	Page 10-11
Patient Perspective	12	The patient should share their perspective in one to two paragraphs on the treatment(s) they received	N/A
Informed Consent	13	Did the patient give informed consent? Please provide if requested	Yes ☑ No ☐
